# Methodology Matters: Comparing Approaches for Defining Persistent Symptoms after Mild Traumatic Brain Injury

**DOI:** 10.1089/neur.2021.0028

**Published:** 2021-12-13

**Authors:** Migle Karaliute, Simen B. Saksvik, Hanne Smevik, Turid Follestad, Cathrine Einarsen, Anne Vik, Asta K. Håberg, Grant L. Iverson, Toril Skandsen, Alexander Olsen

**Affiliations:** ^1^ Department of Psychology, Norwegian University of Science and Technology (NTNU), Trondheim, Norway.; ^2^ Department of Neurology, St. Olavs Hospital, Trondheim University Hospital, Trondheim, Norway.; ^3^ Department of Physical Medicine and Rehabilitation, and St. Olavs Hospital, Trondheim University Hospital, Trondheim, Norway.; ^4^ Department of Clinical and Molecular Medicine, Faculty of Medicine and Health Sciences, and Norwegian University of Science and Technology (NTNU), Trondheim, Norway.; ^5^ Department of Neuromedicine and Movement Science, Norwegian University of Science and Technology (NTNU), Trondheim, Norway.; ^6^ Department of Neurosurgery, St. Olavs Hospital, Trondheim University Hospital, Trondheim, Norway.; ^7^ Department of Physical Medicine and Rehabilitation, Harvard Medical School; Spaulding Rehabilitation Hospital and Spaulding Research Institute; MassGeneral Hospital for Children Sports Concussion Program; & Home Base, A Red Sox Foundation and Massachusetts General Hospital Program, Charlestown, Massachusetts, USA.

**Keywords:** concussion, outcome, post-concussion syndrome, prognosis, Rivermead Post Concussion Symptom Questionnaire, The British Columbia Post-Concussion Symptom Inventory

## Abstract

Some people experience persistent post-concussion symptoms (PPCS) after mild traumatic brain injury (mTBI). A meaningful clinical classification and scientific progress are hampered by a lack of consensus regarding the phenomenology, assessment, and operationalization of PPCS. Here we demonstrate and evaluate how the methodology used to assess and define persistent symptoms after mTBI influences PPCS as a binary outcome. We present empirical data from 15 classification methods reflecting procedures found in the literature and clinical practice. In total, 221 patients with mTBI, 73 patients with orthopedic injuries, and 77 community controls were included in the study. The prevalence rate of PPCS in the mTBI group varied between 10% and 47%, depending on the method used to assess and define unfavorable outcome. There was generally low positive agreement between the different methods; even the two methods yielding the most similar prevalence rates (89.2% overall proportion agreement) agreed on less than half (45.5% positive agreement) of the PPCS cases. Using a liberal but not uncommon threshold for symptom severity, there was a considerable misclassification rate of PPCS in both comparison groups. Our results highlight the importance for researchers to be aware of the limitations of using binary approaches for classification of PPCS. The poor agreement between methods should be considered when (1) interpreting the heterogeneity in the existing PPCS literature and (2) developing new improved methods. An empirically informed consensus regarding classification of PPCS should be a priority for the research community.

## Introduction

Some people who sustain a mild traumatic brain injury (mTBI) experience persistent post-concussion symptoms (PPCS) for months^1–3^ or years.^4,5^ The biopsychosocial model^6^ implies that PPCS can be influenced or caused by a diverse set of factors, including genetics,^7^ sex, age, stressful life events,^8^ pre-injury physical health or psychological issues,^9^ injury related factors,^9^ the severity of acute or subacute post-concussion symptoms,^10^ post-injury anxiety,^11^ traumatic stress,^10^ or the development of post-injury sleep-wake disturbance^12,13^ and depression.^14^ Social psychological factors, such as expectations,^15^ diagnosis threat,^16^ the good-old-days bias,^17^ and secondary gain^18^ have also been associated with symptom reporting.

Despite the recognized phenomenological complexity of PPCS,^19,20^ in research, its occurrence is often applied as a simple binary outcome. At present, however, there is no consensus on how such an unfavorable outcome after mTBI should be defined and measured.^21,22^ In the literature, this is reflected by the use of many different assessment measures and highly variable definitions regarding the severity threshold, and whether symptoms need to be present across a range of domains, or simply occur at a certain frequency to define the outcome. This variability in defining unfavorable outcome after mTBI is likely an important reason why the observed occurrence of PPCS varies between 6%^23^ and 46%^10,24^ across different studies.

Symptoms after mTBI are typically grouped into symptom categories consisting of somatic, emotional, cognitive, and/or sleep-wake problems. As an example, the World Health Organization (WHO) *The International Classification of Diseases* 10th edition (ICD-10) Diagnostic Criteria for Research^25^ (“Green Book”) requires symptoms to be present in at least three of six different categories to fulfill the diagnosis of post-concussional syndrome (F.07.2). The WHO ICD-10 Diagnostic Guidelines (“Blue Book”), however, only requires that three symptoms are present regardless of category.^26^ Moreover, the ICD-10 criteria and other approaches do not specify any threshold of intensity or frequency for a symptom to be denoted as present. Challenges linked to the lack of pre-defined and validated thresholds have been highlighted in studies demonstrating how a change in cutoff may alter the estimated prevalence of PPCS considerably.^19,27^

Complicating matters further is the fact that post-concussion symptoms are not specific to mTBI. Post-concussion–like symptoms are reported by persons with no history of head trauma—e.g., in persons with chronic pain, depression, anxiety, post-traumatic stress disorder (PTSD) as well as in the general population.^28–30^ Despite previous efforts to evaluate different definitions of PPCS by specifically using the Rivermead Post Concussion Symptoms Questionnaire (RPSQ),^27^ we lack empirical evaluation of a broader selection of methods, which also includes relevant comparison groups without head injury. The latter is important for providing indications on how the specificity of PPCS varies with the different definitions and thresholds used.

Here we aim to demonstrate and evaluate to what extent the methodology used to assess and define PPCS influences outcome classification after mTBI. The study was performed using data from a representative and well-characterized cohort of patients with mTBI and two comparison groups: community controls and trauma controls—i.e., patients with mild orthopedic injuries.^31^ All patients were assessed three months after their injury. Community controls were assessed three months after inclusion in the study.

Extending previous studies,^27^ we compared a range of methods derived primarily from two well-established assessment measures: the British Columbia Post-Concussion Symptom Inventory (BC-PSI)^30^ and the RPSQ.^32^ Given the lack of consensus in defining the relevant burden of post-concussive problems,^19,21,22^ the different methods were evaluated using two different symptom intensity level cutoffs and different degrees of adherence to symptom categories according to the ICD-10 Diagnostic Criteria for Research.^25^ In total, we present data from 15 methods that reflect approaches found in the research literature and clinical practice that have been used previously to define PPCS.

## Methods

### Participants

The participants were part of the Trondheim MTBI Follow-up Study that has been described in greater detail elsewhere.^31^ Briefly, patients were recruited prospectively from two emergency departments (ED): St. Olav's Hospital, Trondheim University Hospital (a Norwegian regional level 1 trauma center) and Trondheim Municipal Emergency clinic (a general practitioner run outpatient clinic open 24/7 and located at the hospital). Patients were between 16 and 60 years old and had sustained a TBI^33^ that was categorized as mTBI according to the WHO Task Force definition^34^: Glasgow Coma Scale (GCS) score of 13–15 at presentation to the ED, loss of consciousness (LOC), if present, <30 minutes, and duration of post-traumatic amnesia (PTA) <24 h.

Exclusion criteria were: (1) non-residency in Norway or non-fluency in the Norwegian language, (2) pre-injury severe psychiatric or somatic disease, or drug abuse that could interfere with follow- up; (3) history of complicated mild, moderate, or severe TBI or other neurological conditions with visible brain pathology or known cognitive deficits; (4) history of uncomplicated mTBI during the last three months before the injury of interest; (5) presenting >72 h after the initial trauma; or (6) presence of other concurrent major trauma, such as spinal cord injury, severe fractures, or internal injuries.

Two control groups were included in the study—one community control group (CC) and a trauma control (TC) group. The CC group consisted of a convenience sample of employees and students at the university hospital as well as friends and family members of employees and patients with mTBI. The CCs were excluded if they received any treatment for psychiatric disorders and if they met any of the mTBI group exclusion criteria. The TC group consisted of patients with orthopedic injuries recruited from the same EDs as the mTBI group. The same exclusion criteria were used for the TC group as for patients with mTBI and, in addition, TCs were not included if they had head, neck, or dominant upper extremity injuries.

A total of 378 patients with mTBI, 82 TCs, and 83 CCs were included in the Trondheim MTBI follow-up study. To allow direct comparison between different classification methods, only individuals who had complete data for all the selected outcome measures at three months after injury were included. Consequently, this study included 221 participants from the mTBI group, 73 from the TC group, and 77 from the CC group in the final analyses. There were no statistically significant differences in age, sex, or completed education between any of the groups. Demographic and injury-related data are presented in Table 1.

**Table 1.** Demographics and Clinical Characteristics of the Mild Traumatic Brain Injury Group, the Trauma Control Group, and the Community Control Group

Demographic/clinical characteristics	mTBI group	Trauma controls	Community controls	*p*
	(*n* = 221)	(*n* = 77)	(*n* = 73)	
Median age (IQR; 25% - 75%)	26.6 (21.2–45.4)	27.5 (21.3–46.3)	28.7 (22.9–44.2)	0.839^a^
Female sex	36.2%	39.7%	39.0%	0.384^b^
Median years of education (IQR; 25%–75%)	13.0 (12.0–16.0)	14.0 (12.0–16.0)	13.0 (12.0–16.0)	0.423^a^
Injury mechanism				
Fall	38.9%	31.1%		
Bicycle	20.4%	9.6%		
Sports accidents	14.0%	37.0%		
Violence	9.5%	1.4%		
Motor vehicle accidents	10.0%	4.1%		
Hit object	6.3%	6.8%		
Other/unknown	0.9%	11.0%^c^		
GCS score				
13	2.3%			
14	12.7%			
15	76.5%			
LOC (%)				
Yes	46.6%			
No	18.6%			
Unknown	34.8%			
PTA (%)				
<1 h	72.4%			
1–24 h	27.6%			
Intracranial findings on CT				
Yes	6.3%			
No	74.2%			
No CT	19.5%			
Level of care				
Not admitted to the hospital	67.9%	86.3%		
Clinical observation <24 h	17.2%	0.0%		
Admitted to the hospital >24 h	14.9%	13.7%		

mTBI, mild traumatic brain injury; IQR, interquartile range (25th–75th percentile); GCS, Glasgow Coma Scale, LOC, loss of consciousness; PTA, post-traumatic amnesia.

^a^ Kruskal-Wallis test; ^b^Pearson chi-square test; ^c^Sharp injuries, such as cuts, are included here for the trauma control group.

The study was approved by the Regional Committees for Medical and Health Research Ethics in Central Norway (REK 2013/754) and performed according to the Helsinki Declaration. Informed consent was obtained from all participants and the participant's guardian if the participant was younger than 18 years.

### Assessment measures

All participants underwent a structured interview either in person or over the telephone at three months after injury (mTBI and TC groups) or inclusion in the study (for CC group). Mimicking the first encounter in a typical clinical examination, patients were first asked a relatively open question to assess presence of symptoms (see Simplified PPCS question below). We then administered the BC-PSI.^30^ After the structured interview, the patients completed the RPSQ^32^ that had either been sent out by mail or was delivered at a face-to-face visit. The CCs only completed the BC-PSI because both the simplified question and the RPSQ pre-suppose that an injury has occurred.

#### Simplified PPCS question

Participants in the mTBI and the TC group were asked to respond to the question: “Do you currently notice anything at all related to your (head) injury?” by using the three alternatives: yes, no, or unsure. This question has not been described in the published research literature and would not typically be used by itself as a clinical definition of PPCS, but was included because it represents a screener question that mimics the first encounter in a typical clinical examination.

#### BC-PSI

The BC-PSI was developed originally to align with the ICD-10 Research Diagnostic Criteria for the Postconcussional syndrome diagnosis, and this measure has sound psychometric properties.^29,30^ We used a Norwegian version that was developed in collaboration with a translator and the original author (GLI). The BC-PSI contains 16 items that assess 13 different symptoms (e.g., headaches, irritability, poor concentration, sleep problems, etc.), and three life problems (alcohol tolerance, worrying about symptoms, and concern about having damage to your brain). The BC-PSI's items as well as the categorization of those items according to ICD-10 symptom categories are illustrated in Table 2b.

**Table 2.** *The International Classification of Diseases* 10th Edition Symptom Categories and Inventory Items

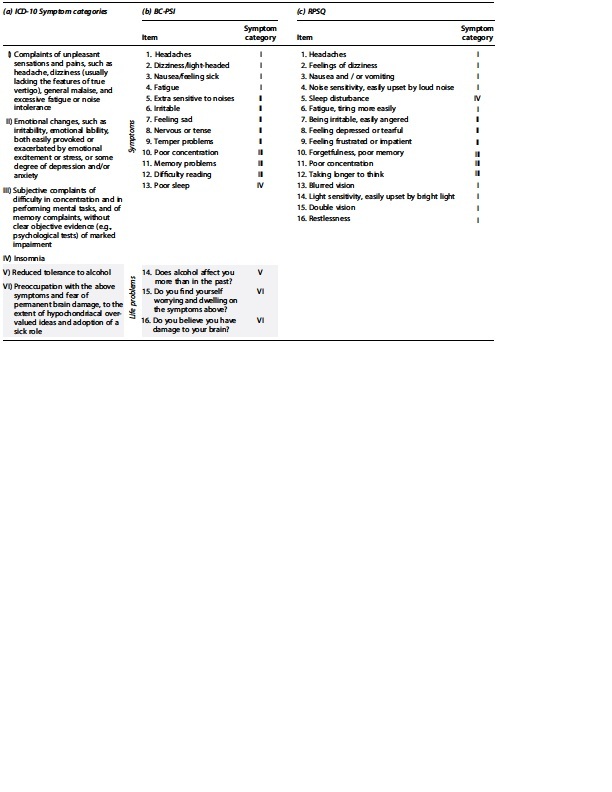						

This table shows the (**a**) *International Classification of Diseases* (ICD-10) *Classification of Mental and Behavioural Disorders,* Diagnostic criteria for research symptom categories^25^ and the corresponding categorization of items on the (**b)** British Columbia Post-Concussion Symptom Inventory **(**BC-PSI) and (**c)** Rivermead Post Concussion Symptom Questionnaire (RPSQ). Item categorization according to the ICD-10 symptom categories are displayed in roman numerals. The light grey color indicates ICD-10 symptom categories V and VI. The BC-PSI classifies the latter as “life problems,” and symptoms from these two categories are not included in the RPSQ. According to the ICD-10 diagnostic criteria for research, patients must report symptoms from at least three of the six listed symptom categories to fulfill the criteria for a post-concussion syndrome diagnosis. See Table 3 for details on how symptom categories and inventory items were used in the different persistent post-concussion symptoms classification methods used in this study.

**Table 3.** Fifteen Methods for Classification of Persistent Post-Concussion Symptoms

Assessment tool	Method for classification of PPCS or PPCS-like cases		Abbreviated name	Symptom severity cutoff
**1: Simplified PPCS question**	**1**	Do you currently notice anything at all related to your (head) injury?	*Simplified PPCS question*	Answering “yes” (out of possible answers “yes,” “no,” “unsure”)
**2 – 10: British Columbia Post Concussion Symptom Inventory (BC-PSI)**	2^*^ 3^**^	Endorsing symptoms from at least 3 out of 6 ICD-10 symptom categories (I–VI).	*BC-PSI, 3/6 ICD-10*	^*^) Mild or greater (item score ≥1; life problem score ≥2). ^**^) Moderate or greater (item score ≥3; life problem score ≥4)
	4^*^ 5^**^	Endorsing symptoms from at least 3 out of 4 core ICD-10 symptom categories (I – IV).	*BC-PSI, 3/4 ICD-10*	
	6^*^ 7^**^	Endorsing 3 or more different symptoms / life problems from all 16 items.	*BC-PSI, 3/16 items*	
	8^*^ 9^**^	Endorsing 3 or more different symptoms from items 1–13 (excluding life problems).	*BC-PSI, 3/13 items*	
	**10**	Having a total score of 13 or more, calculated from item scores on items 1–13 (excluding life problems).	*BC-PSI, total score ≥ 13*	*N/A*
**11 – 15: Rivermead Post Concussion Symptoms Questionnaire (RPSQ)**	11^*^ 12^**^	Endorsing symptoms from at least 3 out of 4 core ICD-10 symptom categories (I – IV).	*RPSQ, 3/4 ICD-10*	^*^) Mild or greater (item score ≥2) ^**^) Moderate or greater (item score ≥3)
	13^*^ 14^**^	Endorsing at least 3 different symptoms (items) from all 16 items.	*RPSQ, 3/16 items*	
	**15**	Having a total score of 16 or more, calculated from item scores of 2 or higher.	*RPSQ, total score ≥ 16*	Item score ≥2

PPCS, persistent post-concussion symptoms,;ICD-10, *The ICD-10 Classification of Mental and Behavioural Disorders*, Diagnostic criteria for research.^25^ See Table 2 for details on how items from the different inventories (RPQS and BC-PSI) correspond to ICD-10 symptom categories. Abbreviated names for each method (third column) are continued as labels in Table 4 and Figures 2, 3.

**Table 4.** Prevalence of Persistent Post-Concussion or Post-Concussion-Like Cases based on the 15 Different Methods

Method	mTBI group (*n* = 221)	Trauma controls (*n* = 73)	Community controls (*n* = 77)
**1.** Simplified PPCS question, answering “Yes”	27.1 % (60)	64.4% (47)	N/A
**2.** BC-PSI, symptoms from at least 3 out of 6 ICD-10 symptom categories (I–VI), ≥ mild severity	37.8% (83)	30.1% (22)	24.7% (19)
**3.** BC-PSI, symptoms from at least 3 out of 6 ICD-10 symptom categories (I–VI), ≥ moderate severity	16.2% (36)	8.2% (6)	1.3% (1)
**4.** BC-PSI, symptoms from at least 3 out of 4 core ICD-10 symptom categories (I-IV), ≥ mild severity	26.7% (59)	20.5% (15)	16.9% (13)
**5.** BC-PSI, symptoms from at least 3 out of 4 core ICD-10 symptom categories (I-IV), ≥ moderate severity	10.0% (22)	1.4% (1)	0.0% (0)
**6.** BC-PSI, 3 or more symptoms/life problems (from items 1–16), ≥ mild severity	47.1% (104)	34.2% (25)	31.2% (24)
**7.** BC-PSI, 3 or more symptoms/ life problems (from items 1–16), ≥ moderate severity	42.5% (94)	30.1% (22)	27.3% (21)
**8.** BC-PSI, 3 or more symptoms (from items 1–13), ≥ mild severity	19.5% (43)	9.6% (7)	1.3% (1)
**9.** BC-PSI, 3 or more symptoms (from items 1–13), ≥ moderate severity	15.8% (35)	4.1% (3)	0.0% (0)
**10.** BC-PSI, total score ≥13	18.1% (37)	6.8% (5)	1.3% (1)
**11.** RPSQ, symptoms from at least 3 out of 4 core ICD-10 symptom categories (I–IV), ≥ mild severity	26.7% (59)	8.2% (6)	N/A
**12.** RPSQ, symptoms from at least 3 out of 4 core ICD-10 symptom categories (I–IV), ≥ moderate severity	10.0% (22)	2.7% (2)	N/A
**13.** RPSQ, 3 or more symptoms (from items 1–16), ≥ mild severity	33.5% (74)	12.3% (9)	N/A
**14.** RPSQ, 3 or more symptoms (from items 1–16), ≥ moderate severity	16.3% (36)	4.1% (3)	N/A
**15.** RPSQ, total score ≥16	18.6% (41)	2.7% (2)	N/A

mTBI, mild traumatic brain injury; N/A, not applicable; PPCS, persistent post-concussion symptoms; ICD, *International Classification of Diseases;* BC-PSI, British Columbia Post-Concussion Symptom Inventory; RPSQ, Rivermead Post Concussion Symptom Questionnaire; ICD-10; *The ICD-10 Classification of Mental and Behavioural Disorders,* Diagnostic criteria for research.^25^

For BC-PSI, participants were asked to rate their experience with each symptom/life problem over the past two weeks—including the day of assessment. Symptom experience (items 1–13) was rated on both frequency and intensity using six-point Likert-scales: Frequency: (0 = Not at all, 1 = 1–2 times, 2 = Several times, 3 = Often, 4 = Very often, and 5 = Constantly). Intensity: (0 = Not at all, 1 = Very mild problem, 2 = Mild problem, 3 = Moderate problem, 4 = Severe problem, and 5 = Very severe problem). Experience of life problems (item 14–16), was rated using a five-point Likert-scale (1 = Not at all; 3 = Somewhat; 5 = Very much).

To derive item scores for items 1–13, frequency and intensity ratings for each symptom were multiplied into an intermediary product score, which was then transformed to item scores in the following way: 0–1 transformed to 0; 2–3 transformed to 1; 4–6 transformed to 2; 8–12 transformed to 3, and ≥15 transformed to 4. Item scores of ≥1 were categorized as mild or greater symptom endorsement, and scores of ≥3 as moderate or greater symptom endorsement. For life problems (item 14–16), the five-point rating was used to rate symptom severity directly. Scores of ≥2 were defined as mild or greater endorsement, and ≥4 as moderate or greater endorsement.

#### RPSQ

The RPSQ is a well-established and frequently used questionnaire in mTBI research.^32^ A Norwegian version approved by the original author was used in the present study. The RPSQ presupposes that the respondent has sustained an injury and was therefore not completed by the CC group. The questionnaire consists of 16 items describing different symptoms, and respondents are asked to indicate the severity of each symptom experienced the last 24 hours compared to before the injury. The RPSQ and the categorization of items according to ICD-10 symptom categories are illustrated in Table 2c. Note that the RPSQ does not include items corresponding to ICD-10 symptom categories V and VI.

Participants were asked to rate each item using a five-point Likert scale, yielding a direct item score: (0 = Not experienced at all; 1 = No more of a problem; 2 = Mild problem; 3 = Moderate problem; and 4 = Severe problem). Item scores of ≥2 were categorized as mild or greater symptom endorsement, and scores of ≥3 as moderate or greater symptom endorsement. In accordance with previous studies, item scores of 1 were not included in any analyses because they represent “no more of a problem” compared with before the injury.^35,36^

### Classification methods

We applied four different overarching approaches leading to 15 methods to achieve a binary PPCS (-like) classification in our sample. Where applicable, methods were separated based on their symptom severity level threshold (mild or greater/moderate or greater). Details can be found in Table 2 and Table 3. Briefly, our first approach aimed to mimic a typical clinical encounter asking a relatively open question about symptom experience (Simplified PPCS question; method 1). Our second approach was based on using the WHO ICD-10 Research Diagnostic Criteria (Green Book)^25^ as a framework for categorization of PPCS.

In addition to requiring that the symptoms have a temporal relationship to the head trauma and are not better explained by other conditions, these diagnostic criteria require that symptoms from at least three of the six listed symptom categories are present to qualify for diagnosis. The ICD-10 symptom categories and corresponding items on the BC-PSI and RPSQ are illustrated in Table 2. The BC-PSI assesses all six ICD-10 symptom categories (method 2-3), but it is commonly used without the items assessing life problems corresponding to symptom categories V-VI (method 4-5).^37^ The latter approach is more comparable to the RPSQ, which only assesses ICD-10 symptom categories I–IV (method 11-12).^36,38^

Our third approach was included to reflect more general procedures (e.g., as in the ICD-10 Blue Book and similar) that focus on the number of different symptoms (e.g., three or more) reported from those listed in each inventory regardless of symptom category (BC-PSI—method 6-9; RPSQ—method 13-14).^1,39^ Finally, our fourth and final approach was to apply commonly used total score cutoffs for the BC-PSI (cutoff ≥13; method 10)^40^ and the RPSQ (cutoff ≥16; method 15).^41^

### Statistical analyses

The IBM SPSS 25 (IBM, Armonk, NY) was used for statistical analyses. Categorical variables are described using the frequency (*n*) and percentage (%), and continuous data (age and education) are presented using median with interquartile range (IQR). To evaluate demographic differences between the mTBI group and the CCs and TCs, we used the Pearson chi-square tests for categorical data (e.g., sex), and the Kruskal-Wallis test for continuous data (i.e., age and education). The *p* values <0.05 were considered statistically significant.

For each of the 15 different methods used to assess post-concussion symptoms, the results were summarized by the number and the percentage of participants categorized as having PPCS. Moreover, the overall proportion agreement, as well as the negative and the positive proportion agreement between any two methods, were estimated and presented as percentages. The Cohen's kappa was calculated for each pair of methods. We considered kappa values between 0–0.20 as an expression of poor agreement, 0.21–0.40 as fair agreement, 0.41–0.60 as moderate agreement, 0.61–0.80 as good agreement, and 0.81–1 as very good agreement.^42^

## Results

### Study population

Demographics and clinical characteristics of the mTBI group, the TC group, and the CC group can be found in Table 1. The samples were similar in sex distribution, age, and education. Most participants with mTBI had GCS scores of 15 (77.0%) in the ED and PTA duration estimates of <1 h (72.4%).

### Prevalence of PPCS across different classification methods

The prevalence of PPCS or PPCS-like cases for the 15 different methods is presented in Table 4. When we asked the patients the Simplified PPCS question, 27.6% of the mTBI group responded yes, whereas this was the case for 64.4% in the TC group. The prevalence of mild or greater PPCS/PPCS-like cases varied from 26.7% to 47.1% in the mTBI group, from 8.2% to 34.2% in the TC group, and from 16.9% to 31.2% in the CC group. The prevalence of moderate or greater PPCS/PPCS-like cases varied from 10.0% to 19.5% in mTBI group, from 1.4% to 9.6% in the TC group, and from 0% to 1.3% in the CC group (Table 4).

### Agreement between different methods in defining PPCS

**FIG. 1.**
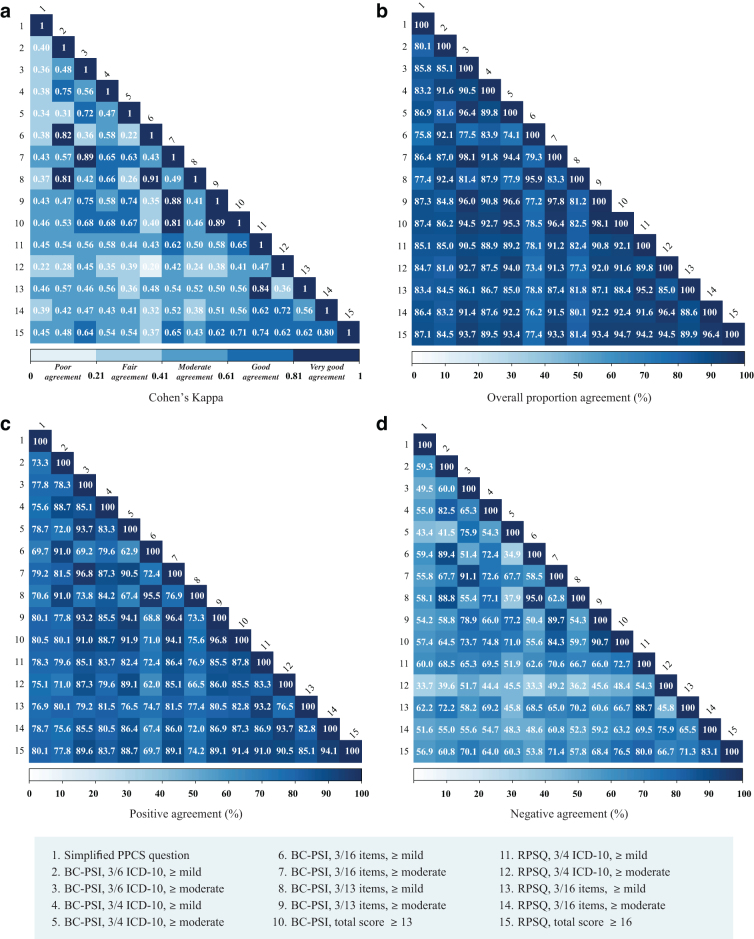
Agreement between methods. Cohen's kappa (κ) values (**a**), overall proportion agreement (**b**), positive agreement (**c**), and negative agreement (**d**) for all 15 methods. See Table 3 for detailed descriptions of each method. Darker colors indicate higher agreement values. The κ values vary from 0 to 1 and were categorized according to Altman (1990).^42^ PPCS, persistent post-concussion symptoms; BC-PSI, British Columbia Post-Concussion Symptom Inventory; ICD-10, *The International Classification of Diseases* (*ICD-10) Classification of Mental and Behavioural Disorders* Diagnostic criteria for research.^25^ RPSQ, Rivermead Post Concussion Symptom Questionnaire.

The values for four different measures of agreement between the 15 methods are presented in Figure 1. The Simplified PPCS question and the other methods examined had an overall proportion agreement ranging from 69.7% to 80.5%, a positive agreement ranging from 33.7% to 62.2%, and a negative agreement ranging from 75.8% to 87.4%. The Cohen's kappa values ranged from 0.22 to 0.46, indicating fair to moderate agreement. The observed overall proportion agreement between the different methods based on the BC-PSI and RPSQ varied from 62.0% to 91.4%. Positive agreement varied from 33.3% to 76.5% and negative agreement varied between 73.4% and 94.7%.

**FIG. 2.**
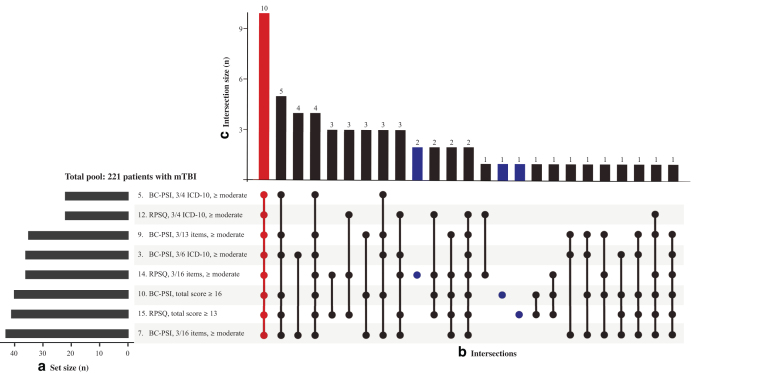
Overlap in persistent post-concussion symptoms (PPCS) classification using selected methods. This upset plot shows the overlap in PPCS classification for the eight methods that are most conservative—i.e., using a symptom severity threshold of moderate or greater or using total scores from British Columbia Post-Concussion Symptom Inventory (BC-PSI) and Rivermead Post Concussion Symptom Questionnaire (RPSQ). See Table 3 for detailed descriptions of each method. The plot has three elements: (**a**) set size, showing the overall number of patients classified with PPCS using a certain method, (**b**) intersections, as displayed in a graphical presentation indicating combinations of methods that classify unique sets of patients, and (**c**) intersection size, which is depicted as a bar chart showing the number of PPCS cases uniquely classified using a certain combination of methods. Highlighted in red color: in a total pool of 221 patients, only 10 patients were classified as having PPCS across all different methods. Highlighted in blue color: a total of four cases were classified with PPCS using only one method alone (no overlap between methods). mTBI, mild traumatic brain injury.

Not surprisingly, the lowest agreement was observed between the most conservative method (method 12; RPSQ using ICD-10 categories at moderate or greater level) and the most liberal method (method 6; BC-PSI endorsing at least 3 symptoms/life problems from all 16 items, at mild or greater level). For the most conservative methods (using a symptom threshold of moderate or higher, or total scores from BC-PSI and RPSQ), only 10 patients were classified with PPCS across all methods (Fig. 2).

The highest overall proportion agreement was observed for the methods that were based on the BC-PSI and RPSQ and required endorsement of symptoms from at least three of four ICD-10 symptom categories at a moderate or greater severity level (89.1%). These methods classified the same number of PPCS cases in the mTBI group (*n* = 22), but the methods agreed on fewer than half of those participants (*n* = 10) resulting in a positive agreement of 45.5%, negative agreement of 94.0%, and κ = 0.39 (Fig. 1; Table 5).

**Table 5.** Agreement between Selected Approaches based on the British Columbia Post-Concussion Symptom Inventory and Rivermead Post Concussion Symptom Questionnaire

**a) Mild or greater**			
κ: 0.58 Overall agreement: 83.7% Positive agreement: 69.5% Negative agreement: 88.9%		**4**. BC-PSI, 3/4 ICD-10	
		PPCS-	PPCS+
**11.** RPSQ, 3/4 ICD-10	PPCS-	144	18
	PPCS+	18	41
**b) Moderate or greater**			
κ: 0.39 Overall agreement: 89.1% Positive agreement: 45.5% Negative agreement: 94.0%		**5**. BC-PSI, 3/4 ICD-10	
		PPCS-	PPCS+
**12.** RPSQ, 3/4 ICD-10	PPCS-	187	12
	PPCS+	12	10
**c) Mild or greater**			
κ: 0.52 Overall agreement: 77.4% Positive agreement: 70.2% Negative agreement: 81.8%		**8**. BC-PSI, 3/13 items	
		PPCS-	PPCS+
**13.** RPSQ, 3/16 items	PPCS-	112	35
	PPCS+	15	59
**d) Moderate or greater**			
κ: 0.51 Overall agreement: 86.9% Positive agreement: 59.2% Negative agreement: 92.2%		**9**. BC-PSI, 3/13 items	
		PPCS-	PPCS+
**14.** RPSQ, 3/16 items	PPCS-	171	14
	PPCS+	15	21

The tables show agreement between selected approaches based on the British Columbia Post-Concussion Symptom Inventory (BC-PS) and Rivermead Post Concussion Symptom Questionnaire (RPSQ) using the four core ICD-10 categories (category I–IV). Tables **a)** and **b)** show agreement when requiring symptoms from at least three categories (**a** and **b**), and tables **c)** and **d)** show agreement when requiring endorsement of at least three symptoms regardless of symptom category (**c** and **d**). See Table 2 for full version of abbreviated names for the different methods. Tables **a)** and **c)** show agreement at using a symptom severity level threshold of mild or greater, and **b)** and **d)** show agreement using a symptom severity level threshold of moderate or greater. Although overall agreement is relatively high, positive agreement and Cohen's kappa (κ) values are generally low, indicating considerable dissimilarity between the persistent post-concussion symptoms (PPCS) cases identified by the two inventories. ICD-10, *The ICD-10 Classification of Mental and Behavioural Disorders,* Diagnostic criteria for research.^25^

## Discussion

In this prospective study of patients with mTBI, the estimated occurrence of PPCS three months after injury varied between 10.0% and 47.1%, depending on the method used to assess and define unfavorable outcome. Overall, the different methods exhibited only modest agreement at best. Notably, the two methods yielding the most similar prevalence rates (89.2% overall proportion agreement) only had a positive agreement of 45.5%, meaning that the respective methods disagreed in more than half of the PPCS cases. The methods using a criterion of mild or greater symptom severity cutoff yielded a two-to-five–fold larger prevalence rate of PPCS than the methods using a moderate or greater symptom severity cutoff. Using a mild or greater symptom severity cutoff yielded a considerable proportion of PPCS-like cases in the two control groups across methods, especially in TCs.

The number of persons misclassified in the CC and TC groups was substantially fewer when raising the threshold to include only persons endorsing moderate or greater symptom severity. This suggests that at least a subgroup of patients with more severe PPCS are experiencing symptoms that are phenomenologically different from most people classified as PPCS-like cases in the control groups.^19,27^ Because of the lack of consensus or gold standard for defining PPCS,^21,22^ we cannot determine which of our included methods perform better relative to an *a priori* prediction. Nonetheless, the analyses presented here are informative for researchers planning future studies and data analyses.

The largest source of variance in PPCS classification in this study could be attributed to symptom severity thresholding, but there was also considerable disagreement between methods because of differences in symptom category and frequency, as well as other factors associated with the design of the inventories included.

Our study extends previous work by examining the classification properties of both the RPSQ^27^ and the BC-PSI, and evaluating the agreement between methods based on these widely used symptom inventories. The BC-PSI and RPSQ provided similar prevalence rates of PPCS, but there was at most moderate agreement (Cohen's kappa) between the two inventories, meaning that they identify overlapping but also distinct subgroups of patients. This highlights the importance of looking beyond prevalence rates when considering the use and interpretation of different inventories and classification methods.

The RPSQ and BC-PSI have considerable, but not complete, overlap in the type of symptoms included, but importantly they differ in the time for which symptoms are endorsed. For the BC-PSI, participants are asked to indicate whether they have experienced symptoms during the last two weeks, whereas the RPSQ assesses symptoms experienced within the last 24 h. Moreover, the item scores derived from BC-PSI are a product of the frequency and intensity of a symptom, but the RPSQ queries to what degree the symptom represents a problem. The PPCS may fluctuate, within and between days and weeks. Assessing symptoms from different periods may therefore explain some of the discrepancy between the two instruments.

It is possible that the BC-PSI identifies certain individuals compared with the RPSQ because it queries symptoms over the past two weeks versus the past 24 h. On the other hand, the RPSQ might have a strength in capturing day-to-day variations, if used repeatedly, but this was not possible to evaluate using the single-point evaluation in our study. The different periods assessed by the inventories may also result in different test-retest reliability depending on the period of interest. Although not addressed in our study, a recent study of PPCS in adolescents shows that self-report of symptoms is only modestly reliable over a four-month period, even in a healthy control.^43^

Another key difference between the two inventories is that in the RPSQ, participants are asked specifically to rate their symptoms in comparison with how they felt before they were injured, whereas this is not the case for the BC-PSI. Because of this, one may speculate that the BC-PSI will yield a greater rate of misclassification of PPCS in control groups^30^ and that the RPSQ is more likely to identify true cases in the mTBI group. Some support for this can be found in our data, considering that the prevalence rate of PPCS-like cases in the TC group using methods based on the BC-PSI was up to twofold of that when using the RPSQ. Because the total prevalence rate in the mTBI group was similar across the two inventories using comparable criteria, however, it is still unclear whether there is a relevant difference within the mTBI patient group.

Individual response styles may vary, not only based on the face-value interpretation of the items and instructions included in the inventories, but also with factors such as expectations,^15^ diagnosis threat,^16^ the good-old-days bias,^17^ and secondary gain.^18^ Our study provides useful information for using BC-PSI and RPSQ, but assessment measures and methods that also take into consideration other non-injury–related factors not assessed in these inventories may be necessary to fully grasp the complexity of PPCS classification.

The prevalence and agreement of PPCS varied depending on whether symptoms had to be present across several symptom categories or simply occur at a certain frequency. As expected, methods utilizing fewer possible symptoms/symptom categories in their classification criteria were associated with lower prevalence rates. Interestingly, when we asked the patients a simplified single question about whether they still noticed anything at all related to their injury, 64.4% of the TC group responded yes, whereas this was true for only 27.1% in the mTBI group. This is particularly interesting in that approximately two of three TC patients notice, broadly defined, some lingering issues relating to being injured—a proportion that was higher than the mTBI group.

Somewhat counterintuitive was that the prevalence rate of PPCS using methods based on the RPSQ (which asks specifically about symptoms in comparison with how they felt before they were injured), using a mild or greater symptom level cutoff, was higher than when using the single open question with the mTBI group. This illustrates three interesting points: (1) asking a general question mimicking the first encounter in the clinic at three months after the injury reveals more general symptoms—of any severity level—in a patient group with orthopedic injuries than in patients with mTBI; (2) inventories asking specifically for what is considered post-concussion symptoms yield more PPCS cases in the mTBI group, as expected, than in TCs, and (3) there is likely a subgroup of mTBI patients who only report problems when probed for specific symptoms/symptom domains.

This highlights what is often observed clinically—that many people may benefit from being prompted for their symptoms (i.e., through a questionnaire or structured interview), rather than being asked only open-ended questions. Our study indicates that a structured approach may be especially important for people with mTBI.

A strength of our study is the highly representative sample of mTBI patients^31^ and that we included both TCs and CCs. The incidence of PPCS varies across different clinical settings and recruitment procedures. Our mTBI group was similar to that of other prospective studies in comparable settings, when considering the proportion of patients with positive CT findings (7.9%) and other clinical measures of acute injury severity (Table 1). It should also be noted that the CC group was recruited from the local hospital and personal affiliates and thus may not be representative of the general population.

Our main aim was to investigate specific between-group effects while maintaining control of key demographic variables that are typically associated with reporting physical, cognitive, and emotional symptoms associated with PPCS. Given that there were no between-group differences in age, sex, or education and the groups have been found to be similar regarding a range of psychosocial variables,^44^ there is no obvious reason to believe that recruitment bias is a driver of the results supporting our main conclusion.

The rates of PPCS-like cases in the control groups were relatively small across different methods when we used a pre-defined threshold of moderate or greater symptom level. Yet, the prevalence of post-concussion symptoms in the mTBI group was considerable and comparable to other studies (∼10–20%).^20,27^ This finding lends credibility to patients experiencing severe symptoms after mTBI that they themselves attribute to their injury. In contrast, we found that methods classifying PPCS using a mild or greater symptom severity threshold identified a relatively large proportion of cases in both TCs and CCs.

Our results may be useful for guiding researchers in the selection of a context-appropriate threshold for defining PPCS, particularly in studies without matched TCs for comparison. It is important to acknowledge, however, that methods offering high specificity alone may lack important sensitivity; clinically relevant cases might be lost using too conservative thresholds for classification.

Classification methods using a total scale score cutoff rather than moderate or greater symptom endorsement within pre-defined categories (i.e., capturing individuals with many mild, but less than three moderate symptoms) provided similar PPCS prevalence rates and comparable mTBI specificity. However, considering the agreement scores (Fig. 1) and limited overlap with other methods (Fig. 2), it was clearly demonstrated that approaches using a total scale score cutoff identified yet other partly overlapping but distinct groups of PPCS cases.

Our selection of methods to illustrate the methodological issues with binary PPCS classification reflects approaches previously reported in the research literature and clinical practice, but they are not exhaustive or fully representative of all existing approaches. Other methods exist, and they may have provided somewhat different results than in this study, but there is no obvious reason to believe that agreement would be higher between methods not included here.

Symptom severity thresholding was used as the only criterion to distinguish between some of the classification methods. This has some obvious implications for interpretation of the results, such as the expected lower prevalence rate when a more stringent symptom severity threshold was used. Methods yielding lower prevalence rates will show higher overall proportion agreement and negative agreement, but as shown in our data, positive agreement may still decrease. It is therefore important to carefully consider the different statistical measures of agreement jointly in this context.

Another aspect to be aware of when interpreting the results is that because of the lack of a gold standard for PPCS classification, no *a priori de facto* “true cases” can be defined. This means that, in our analyses, the different methods are not tested against identifying the same true cases, but that each method is allowed to identify unique cases. The lack of a gold standard for PPCS classification also limits direct evaluation of clinical usefulness of the different methods included in this study. For example, increased symptom severity threshold yielded better between-group discrimination, but we still lack information on diagnostic usefulness within the mTBI group (e.g., regarding who may want/need/benefit from clinical follow-up). Requiring moderate or greater symptom reporting results in improved between-group discrimination, but also may come with the price of more “false negative” cases within the mTBI group.

It is also important to keep in mind, of course, that it is easy to differentiate possible persistent symptoms in the mTBI group from post-concussion symptoms in the control groups because the control groups did not sustain injuries to their heads or brains and thus, by definition, cannot have post-concussion symptoms. The difficulty, of course, is determining the extent to which the persistent symptoms in the mTBI group are causally related to the previous injury.

The lack of agreement on how outcome is classified after mTBI has negative consequences for both research and clinical work. First, it complicates comparison of results between different mTBI studies. Second, it challenges the validity and reliability of the results from studies of the prognosis of mTBI and makes it difficult to uncover the etiology of PPCS. An objective marker that is easy to implement and can predict an unfavorable outcome early after mTBI has been sought for a long time by both researchers and clinicians. Blood and cerebrospinal fluid markers, and information from advanced neuroimaging techniques have been investigated as potential prognostic biomarkers for outcome after mTBI. Despite these efforts, we still lack accurate mTBI diagnostic and prognostic biomarkers.

Considering that binary PPCS classification is often used as the main outcome measure in prognostic studies, poor definition and operationalization of the PPCS phenomena is likely one important reason.^45,46^ So far, many different methods for defining post-concussion symptoms have been used, some of which have been highlighted in our work, resulting in considerable uncertainty regarding the number of mTBI patients who will end up needing prolonged healthcare.

Many patients experience personal costs and frustration linked to the controversy of PPCS diagnosis. This controversy is retained by our lack of a standardized approach for diagnostics and outcome classification. Current approaches are insufficient to capture the heterogeneity of symptoms and problems after mTBI as one syndrome (e.g., Postconcussional syndrome, ICD-10).

From a clinical point of view, it is reasonable that patients with mTBIs should receive follow-up regardless of the number of different symptom categories they report. Because no universal treatment standard has proven efficacious for patients with PPCS,^20,47^ clinicians should aim to personalize treatment and rehabilitation according to the most prominent individual symptoms.^48^ Such an approach may focus on targeting post-concussive headache,^49^ fatigue,^50^ or sleep-wake disturbances.^51^ Accordingly, researchers should be aware of the limitations of binary approaches to classifying PPCS; future progress in our field may benefit from focusing on understanding the underlying mechanisms of such individual but transdiagnostic symptoms^52,53^ in the context of mTBI.

## Conclusions

The lack of good agreement between different binary PPCS classification methods in our study was striking but not unexpected.^27^ Our comprehensive approach adds important empirical insight to this well-recognized but unresolved matter in mTBI research.^19,21,22^ Extending and substantiating previous studies, we evaluated a comprehensive selection of 15 methods that have been used previously to identify PPCS cases in research and clinical settings.

By breaking down the results into clearly operationalized classifications, based on two commonly used inventories applied to a large group of representative and well-characterized patients with mTBI and two matched control groups,^31^ we provide insight into the extent the methodology used to assess and define persistent symptoms after mTBI influences outcome classification. Notably, a high burden of post-concussion symptoms was uncommon in persons without head injury, which indicates that a subgroup of patients in the mTBI group with more severe PPCS are experiencing symptoms that are phenomenologically different from most people classified as PPCS-like cases in the control groups.

Our findings are important to consider when interpreting the heterogeneity in the existing research literature and should be appraised when developing new improved methods for classification. New methods may benefit from moving beyond a binary classification of PPCS toward dynamic risk profiles based on identification and classification of individual transdiagnostic symptoms that may serve as more precise targets for treatment. An empirically informed consensus regarding classification of PPCS should be a priority for the research community.

## Abbreviations Used

BC-PSI: British Columbia Post-Concussion Symptom Inventory
CC: community control group
ED: emergency department
GCS: Glasgow Coma Scale
ICD-10: *The International Classification of Diseases* 10th edition
LOC: loss of consciousness
mTBI: mild traumatic brain injury
PPCS: persistent post-concussion symptoms
PTA: post-traumatic amnesia
PTSD: post-traumatic stress disorder
RPSQ: Rivermead Post-concussion Symptoms Questionnaire
TBP: traumatic brain injury
TC: trauma control group
WHO: World Health Organization

## References

[1] Røe, C., Sveen, U., Alvsåker, K., Bautz-Holter, E.J.D. and rehabilitation (2009). Post-concussion symptoms after mild traumatic brain injury: influence of demographic factors and injury severity in a 1-year cohort study. Disabil. Rehabil. 31, 1235–1243. 19116810 10.1080/09638280802532720

[2] Lannsjö, M., Geijerstam, J.L., Johansson, U., Bring, J., and Borg, J. (2009). Prevalence and structure of symptoms at 3 months after mild traumatic brain injury in a national cohort. Brain Inj. 23, 213–219. 19205957 10.1080/02699050902748356

[3] Oldenburg, C., Lundin, A., Edman, G., Nygren-De Boussard, C., and Bartfai, A. (2016). Cognitive reserve and persistent post-concussion symptoms—a prospective mild traumatic brain injury (mTBI) cohort study. Brain Inj. 30, 146–155. 26618716 10.3109/02699052.2015.1089598

[4] Hiploylee, C., Dufort, P.A., Davis, H.S., Wennberg, R.A., Tartaglia, M.C., Mikulis, D., Hazrati, L.N., and Tator, C.H. (2017). Longitudinal study of postconcussion syndrome: not everyone recovers. J. Neurotrauma 34, 1511–1523. 27784191 10.1089/neu.2016.4677 PMC5397249

[5] Åhman, S., Saveman, B., Styrke, J., Björnstig, U., and Stålnacke, B. (2013). Long-term follow-up of patients with mild traumatic brain injury: a mixed-method study. J. Rehabil. Med. 45, 758–764. 24002311 10.2340/16501977-1182

[6] Iverson, G.L. (2019). Network analysis and precision rehabilitation for the post-concussion syndrome. Front. Neurol. 10, 489. 31191426 10.3389/fneur.2019.00489 PMC6548833

[7] Merritt, V.C., and Arnett, P.A. (2016). Apolipoprotein E (APOE) ϵ4 allele is associated with increased symptom reporting following sports concussion. J. Int. Neuropsychol. Soc. 22, 89–94. 26483005 10.1017/S1355617715001022

[8] Van Veldhoven, L.M., Sander, A.M., Struchen, M.A., Sherer, M., Clark, A.N., Hudnall, G.E., and Hannay, H.J. (2011). Predictive ability of preinjury stressful life events and post-traumatic stress symptoms for outcomes following mild traumatic brain injury: analysis in a prospective emergency room sample. J. Neurol. Neurosurg. Psychiatry 82, 782–787. 21242288 10.1136/jnnp.2010.228254

[9] Ponsford, J., Nguyen, S., Downing, M., Bosch, M., McKenzie, J.E., Turner, S., Chau, M., Mortimer, D., Gruen, R.L., Knott, J., and Green, S. (2019). Factors associated with persistent post-concussion symptoms following mild traumatic brain injury in adults. J. Rehabil. Med. 51, 32–39. 30426138 10.2340/16501977-2492

[10] Cnossen, M.C., van der Naalt, J., Spikman, J.M., Nieboer, D., Yue, J.K., Winkler, E.A., Manley, G.T., von Steinbuechel, N., Polinder, S., Steyerberg, E.W., and Lingsma, H.F. (2018). Prediction of persistent post-concussion symptoms after mild traumatic brain injury. J. Neurotrauma 35, 2691–2698. 29690799 10.1089/neu.2017.5486

[11] Dischinger, P.C., Ryb, G.E., Kufera, J.A., and Auman, K.M. (2009). Early predictors of postconcussive syndrome in a population of trauma patients with mild traumatic brain injury. J. Trauma 66, 289–296. 19204499 10.1097/TA.0b013e3181961da2

[12] Theadom, A., Cropley, M., Parmar, P., Barker-Collo, S., Starkey, N., Jones, K., Feigin, V.L., and BIONIC Research Group (2015). Sleep difficulties one year following mild traumatic brain injury in a population-based study. Sleep Med. 16, 926–932. 26138280 10.1016/j.sleep.2015.04.013

[13] Saksvik, S.B., Karaliute, M., Kallestad, H., Follestad, T., Asarnow, R.F., Vik, A., Haberg, A.K., Skandsen, T., and Olsen, A. (2020). The prevalence and stability of sleep-wake disturbance and fatigue throughout the first year after mild traumatic brain injury. J. Neurotrauma 37, 2528–2541. 32460623 10.1089/neu.2019.6898 PMC7698981

[14] Lange, R.T., Iverson, G.L., and Rose, A. (2011). Depression strongly influences postconcussion symptom reporting following mild traumatic brain injury. J. Head Trauma Rehabil. 26, 127–137. 20631632 10.1097/HTR.0b013e3181e4622a

[15] Gunstad, J., and Suhr, J.A. (2001). “Expectation as etiology” versus “the good old days”: postconcussion syndrome symptom reporting in athletes, headache sufferers, and depressed individuals. J. Int. Neuropsychol. Soc. 7, 323–333. 10.1017/s1355617701733061 11311033

[16] Ozen, L.J., and Fernandes, M.A. (2011). Effects of “diagnosis threat” on cognitive and affective functioning long after mild head injury. J. Int. Neuropsychol. Soc. 17, 219–229. 21138607 10.1017/S135561771000144X

[17] Lange, R.T., Iverson, G.L., and Rose, A. (2010). Post-concussion symptom reporting and the “good-old-days” bias following mild traumatic brain injury. Arch. Clin. Neuropsychol. 25, 442–450. 20447932 10.1093/arclin/acq031

[18] Binder, L.M., and Rohling, M.L. (1996). Money matters: a meta-analytic review of the effects of financial incentives on recovery after closed-head injury. Am. J. Psychiary 153, 7–10. 10.1176/ajp.153.1.7 8540596

[19] Waljas, M., Iverson, G.L., Lange, R.T., Hakulinen, U., Dastidar, P., Huhtala, H., Liimatainen, S., Hartikainen, K., and Ohman, J. (2015). A prospective biopsychosocial study of the persistent post-concussion symptoms following mild traumatic brain injury. J. Neurotrauma 32, 534–547. 25363626 10.1089/neu.2014.3339

[20] Polinder, S., Cnossen, M.C., Real, R.G.L., Covic, A., Gorbunova, A., Voormolen, D.C., Master, C.L., Haagsma, J.A., Diaz-Arrastia, R., and von Steinbuechel, N. (2018). A multidimensional approach to post-concussion symptoms in mild traumatic brain injury. Front. Neurol. 9, 1113. 30619066 10.3389/fneur.2018.01113 PMC6306025

[21] Rose, S.C., Fischer, A.N., and Heyer, G.L. (2015). How long is too long? The lack of consensus regarding the post-concussion syndrome diagnosis. Brain Inj. 29, 798–803. 25870975 10.3109/02699052.2015.1004756

[22] Ruff, R.M. (2011). Mild traumatic brain injury and neural recovery: rethinking the debate. NeuroRehabilitation 28, 167–180. 21558623 10.3233/NRE-2011-0646

[23] Spinos, P., Sakellaropoulos, G., Georgiopoulos, M., Stavridi, K., Apostolopoulou, K., Ellul, J., and Constantoyannis, C. (2010). Postconcussion syndrome after mild traumatic brain injury in Western Greece. J. Trauma 69, 789–794. 20938266 10.1097/TA.0b013e3181edea67

[24] Theadom, A., Parag, V., Dowell, T., McPherson, K., Starkey, N., Barker-Collo, S., Jones, K., Ameratunga, S., and Feigin, V.L., BIONIC Research Group. (2016). Persistent problems 1 year after mild traumatic brain injury: a longitudinal population study in New Zealand. Br. J. Gen. Pract. 66, e16–e23. 26719482 10.3399/bjgp16X683161 PMC4684031

[25] World Health Organization. (1993). The ICD-10 Classification of Mental and Behavioural Disorders: Diagnostic criteria for research. World Health Organization (WHO): Geneva.

[26] World Health Organization. (1992). The ICD-10 Classification of Mental and Behavioural Disorders: clinical descriptions and diagnostic guidelines. World Health Organization: Geneva.

[27] Voormolen, D.C., Cnossen, M.C., Polinder, S., Von Steinbuechel, N., Vos, P.E., and Haagsma, J.A. (2018). Divergent classification methods of post-concussion syndrome after mild traumatic brain injury: prevalence rates, risk factors, and functional outcome. J. Neurotrauma 35, 1233–1241. 29350085 10.1089/neu.2017.5257 PMC6909759

[28] Smith-Seemiller, L., Fow, N.R., Kant, R., and Franzen, M.D. (2003). Presence of post-concussion syndrome symptoms in patients with chronic pain vs mild traumatic brain injury. Brain Inj. 17, 199–206. 12623496 10.1080/0269905021000030823

[29] Iverson, G. (2006). Misdiagnosis of the persistent postconcussion syndrome in patients with depression. Arch. Clin. Neuropsychol. 21, 303–310. 16797916 10.1016/j.acn.2005.12.008

[30] Iverson, G.L., and Lange, R.T. (2003). Examination of “postconcussion-like” symptoms in a healthy sample. Appl. Neuropsychol. 10, 137–144. 12890639 10.1207/S15324826AN1003_02

[31] Skandsen, T., Einarsen, C.E., Normann, I., Bjøralt, S., Karlsen, R.H., McDonagh, D., Nilsen, T.L., Akslen, A.N., Håberg, A.K., and Vik, A. (2018). The epidemiology of mild traumatic brain injury: the Trondheim MTBI follow-up study. Scand. J. Trauma, Resusc. Emerg. Med. 26, 34. 29703222 10.1186/s13049-018-0495-0 PMC5921265

[32] King, N.S., Crawford, S., Wenden, F.J., Moss, N.E., and Wade, D.T. (1995). The Rivermead Post Concussion Symptoms Questionnaire: a measure of symptoms commonly experienced after head injury and its reliability. J. Neurol. 242, 587–592. 8551320 10.1007/BF00868811

[33] Menon, D.K., Schwab, K., Wright, D.W., and Maas, A.I. (2010). Position statement: definition of traumatic brain injury. Arch. Phys. Med. Rehabil. 91, 1637–1640. 21044706 10.1016/j.apmr.2010.05.017

[34] Carroll, L.J., Cassidy, J.D., Holm, L., Kraus, J., Coronado, V.G., and WHO Collaborating Centre Task Force on Mild Traumatic Brain Injury. (2004). Methodological issues and research recommendations for mild traumatic brain injury: the WHO Collaborating Centre Task Force on Mild Traumatic Brain Injury. J. Rehabil. Med. Suppl. 43, 113–125. 10.1080/16501960410023877 15083875

[35] Eyres, S., Carey, A., Gilworth, G., Neumann, V., and Tennant, A. (2005). Construct validity and reliability of the Rivermead Post-Concussion Symptoms Questionnaire. Clin. Rehabil. 19, 878–887. 16323387 10.1191/0269215505cr905oa

[36] King, N.S., Crawford, S., Wenden, F.J., Moss, N.E., and Wade, D.T. (1995). The Rivermead Post Concussion Symptoms Questionnaire: a measure of symptoms commonly experienced after head injury and its reliability. J. Neurol. 242, 587–592. 8551320 10.1007/BF00868811

[37] Silverberg, N.D., Panenka, W.J., and Iverson, G.L. (2018). Work productivity loss after mild traumatic brain injury. Arch. Phys. Med. Rehabil. 99, 250–256. 28760573 10.1016/j.apmr.2017.07.006

[38] Dean, P.J.A., O'Neill, D., and Sterr, A. (2012). Post-concussion syndrome: prevalence after mild traumatic brain injury in comparison with a sample without head injury. Brain Inj. 26, 14–26. 22107176 10.3109/02699052.2011.635354

[39] Sterr, A., Herron, K.A., Hayward, C., and Montaldi, D. (2006). Are mild head injuries as mild as we think? Neurobehavioral concomitants of chronic post-concussion syndrome. BMC Neurol. 6, 7. 10.1186/1471-2377-6-7 PMC1382265 16460567

[40] Iverson, G.L., Zasler, N.D., and Lange, R.T. (2006). Post-concussive disorder, in: *Brain Injury Medicine: Principles and Practice*. Demos medical publishing, New York. 2007. pps. 373–405.

[41] Thompson, C., Davies, P., Herrmann, L., Summers, M., and Potter, S. (2016). Approaches to establishing validated cut-off scores on the Rivermead post-concussion symptoms questionnaire (RPQ). Brain Inj. 30, 770.

[42] Altman, D.G. (1990). Practical Statistics for Medical Research. CRC Press, Boca Raton, FL.

[43] Mayer, A.R., Stephenson, D.D., Dodd, A.B., Robertson-Benta, C.R., Pabbathi Reddy, S., Shaff, N.A., Yeates, K.O., van der Horn, H.J., Wertz, C J., Park, G., Oglesbee, S.J., Bedrick, E.J., Campbell, R.A., Phillips, J.P., and Quinn, D.K. (2020). Comparison of methods for classifying persistent post-concussive symptoms in children. J. Neurotrauma 37, 1504–1511. 31964232 10.1089/neu.2019.6805 PMC7307699

[44] Skandsen, T., Stenberg, J., Follestad, T., Karaliute, M., Saksvik, S.B., Einarsen, C.E., Lillehaug, H., Håberg, A.K., Vik, A., Olsen, A., and Iverson, G.L. (2021). Personal factors associated with postconcussion symptoms months after mild traumatic brain injury. Arch/ Phys. Med. Rehabil. 102, 1102–1112. 10.1016/j.apmr.2020.10.106 33127352

[45] Di Battista, A.P., Rhind, S.G., and Baker, A.J. (2013). Application of blood-based biomarkers in human mild traumatic brain injury. Front. Neurol. 4, 44. 23641234 10.3389/fneur.2013.00044 PMC3640204

[46] Bigler, E.D. (2013). Neuroimaging biomarkers in mild traumatic brain injury (mTBI). Neuropsychol. Rev. 23, 169–209. 23974873 10.1007/s11065-013-9237-2

[47] Bergersen, K., Halvorsen, J.O., Tryti, E.A., Taylor, S.I., and Olsen, A. (2017). A systematic literature review of psychotherapeutic treatment of prolonged symptoms after mild traumatic brain injury. Brain Inj. 31, 279–289. 28125305 10.1080/02699052.2016.1255779

[48] Broglio, S.P., Collins, M.W., Williams, R.M., Mucha, A., and Kontos, A.P. (2015). Current and emerging rehabilitation for concussion: a review of the evidence. Clin. Sports Med. 34, 213–231. 25818710 10.1016/j.csm.2014.12.005 PMC4387881

[49] Watanabe, T.K., Bell, K.R., Walker, W.C., and Schomer, K. (2012). Systematic review of interventions for post-traumatic headache. PM R 4, 129–140. 22373462 10.1016/j.pmrj.2011.06.003

[50] Mollayeva, T., Kendzerska, T., Mollayeva, S., Shapiro, C.M., Colantonio, A., and Cassidy, J.D. (2014). A systematic review of fatigue in patients with traumatic brain injury: the course, predictors and consequences. Neurosci. Biobehav. Rev. 47, 684–716. 25451201 10.1016/j.neubiorev.2014.10.024

[51] Sullivan, K.A., Blaine, H., Kaye, S.A., Theadom, A., Haden, C., and Smith, S.S. (2018). A systematic review of psychological interventions for sleep and fatigue after mild traumatic brain injury. J. Neurotrauma 35, 195–209. 28895488 10.1089/neu.2016.4958

[52] Krueger, R.F., and Eaton, N.R. (2015). Transdiagnostic factors of mental disorders. World Psychiatry 14, 27–29. 25655146 10.1002/wps.20175 PMC4329885

[53] Morris, S.E., and Cuthbert, B.N. (2012). Research Domain Criteria: cognitive systems, neural circuits, and dimensions of behavior. Dialogues Clin. Neurosci. 14, 29–37. 22577302 10.31887/DCNS.2012.14.1/smorris PMC3341647

